# Practice patterns of dialysis access and outcomes in patients wait-listed early for kidney transplantation

**DOI:** 10.1186/s12882-020-02080-5

**Published:** 2020-10-02

**Authors:** Raphaëlle Sylvestre, Natalia Alencar de Pinho, Ziad A. Massy, Christian Jacquelinet, Mathilde Prezelin-Reydit, Roula Galland, Bénédicte Stengel, Raphael Coscas, on behalf of the French REIN registry

**Affiliations:** 1 Clinical Epidemiology Team, Paris-Saclay University, Paris-Sud University, UVSQ, CESP, Inserm, Villejuif, France; 2 Division of Vascular Surgery, Ambroise Paré University Hospital, APHP, Boulogne-Billancourt, France; 3 Division of Nephrology, Ambroise Paré University Hospital, APHP, Boulogne-Billancourt, France; 4 Agence de la Biomédecine, Direction Médicale et Scientifique, Boulogne-Billancourt, France; 5 Aurad-Aquitaine, Service Hémodialyse, Saint Denis La Plaine, France; 6 Bordeaux Population Health Research Center, Clinical Investigation Center-Clinical Epidemiology-CIC-1401, University of Bordeaux, INSERM, UMR1219, Bordeaux, France; 7 Calydial – GHM Portes du Sud, Vénissieux, France

**Keywords:** Kidney transplantation, Donor type, Dialysis, Vascular access, Patient survival

## Abstract

**Background:**

Early kidney transplantation (KT) is the best option for patients with end-stage kidney disease, but little is known about dialysis access strategy in this context. We studied practice patterns of dialysis access and how they relate with outcomes in adults wait-listed early for KT according to the intended donor source.

**Methods:**

This study from the REIN registry (2002–2014) included 9331 incident dialysis patients (age 18–69) wait-listed for KT before or by 6 months after starting dialysis: 8342 candidates for deceased-donor KT and 989 for living-donor KT. Subdistribution hazard ratios (SHR) of KT and death associated with hemodialysis by catheter or peritoneal dialysis compared with arteriovenous (AV) access were estimated with Fine and Gray models.

**Results:**

Living-donor candidates used pretransplant peritoneal dialysis at rates similar to deceased-donor KT candidates, but had significantly more frequent catheter than AV access for hemodialysis (adjusted OR 1.25; 95%CI 1.09–1.43). Over a median follow-up of 43 (IQR: 23–67) months, 6063 patients received transplants and 305 died before KT. Median duration of pretransplant dialysis was 15 (7–27) months for deceased-donor recipients and 9 (5–15) for living-donor recipients. Catheter use in deceased-donor candidates was associated with a lower SHR for KT (0.88, 95%CI 0.82–0.94) and a higher SHR for death (1.53, 95%CI 1.14–2.04). Only five deaths occurred in living-donor candidates, three of them with catheter use.

**Conclusions:**

Pretransplant dialysis duration may be quite long even when planned with a living donor. Advantages from protecting these patients from AV fistula creation must be carefully evaluated against catheter-related risks.

## Background

Among patients with end-stage kidney disease, kidney transplantation (KT) is associated with improved survival [1, 2], better quality of life [2, 3], and lower cost [4] than dialysis. Access to KT is a lengthy process, which includes assessing patients’ clinical condition and eligibility for KT, their placement on the national transplant waiting list, and attribution of a graft by the allocation system (managed in France by the *Agence de la Biomedecine)*. Except for preemptively transplanted patients, who account for 4% of the French population treated for end-stage kidney disease [5], most KT candidates need dialysis. Although peritoneal dialysis is a valuable modality for patients awaiting KT [6, 7], hemodialysis is the modality used by far most frequently in this population, accounting for 85% of dialyzed patients awaiting KT in France [8] and in the US [7, 9] and around 67% in Taiwan [6].

Highly variable fractions of overall KT are planned as living-donor KT (LDKT) in different countries: 8% of KT in Finland (3.8 per million population [pmp]), 28% in the US (17.4 pmp), and 57% in the Netherlands (33.6 pmp) [10, 11]. In France, living donation rates have increased significantly over the past decade rising from 5.1 pmp in 2011 to 8.8 pmp in 2016 [12]. LDKT now accounts for 16% of all KT and is associated with better patient and graft outcomes than is deceased-donor KT (DDKT) [2, 13, 14]. Living donation may also provide more timely access to KT, ideally enabling preemptive transplantation and thereby avoiding dialysis and its complications. Nevertheless, most patients require dialysis while on the KT waiting list [15, 16].

Optimizing vascular access is an important challenge in hemodialysis patients. Guidelines have consistently recommended placement of an arteriovenous (AV) fistula over AV graft or central venous catheter (CVC) because the fistula is associated with better outcomes [17, 18]. In response to these recommendations, AV fistula use has increased substantially over the past decade. The frequency of percutaneous and surgical interventions to promote AV fistula maturation and patency has nonetheless increased concurrently [19] Given the burden of an AV fistula for patients and health resources, nephrologists may favor other types of vascular access in patients whose waiting time to KT is expected to be short. Trends in dialysis access strategies used for patients with an expected short wait time to kidney transplantation, i.e., wait-listed early for a living- or deceased-donor kidney, and their impact on outcomes have been poorly evaluated.

We hypothesized that, in adults wait-listed early for KT, for whom pretransplant dialysis duration may thus be expected to be relatively short, there may be a shift from AV access to CVC use. We used data from the national REIN registry to study trends in practice patterns of dialysis access in incident patients wait-listed for KT before or within 6 months after starting dialysis and further assessed the relation of that access strategy with patient survival, according to the planned donor type at KT wait-listing.

## Methods

### Study population

**Fig. 1:**
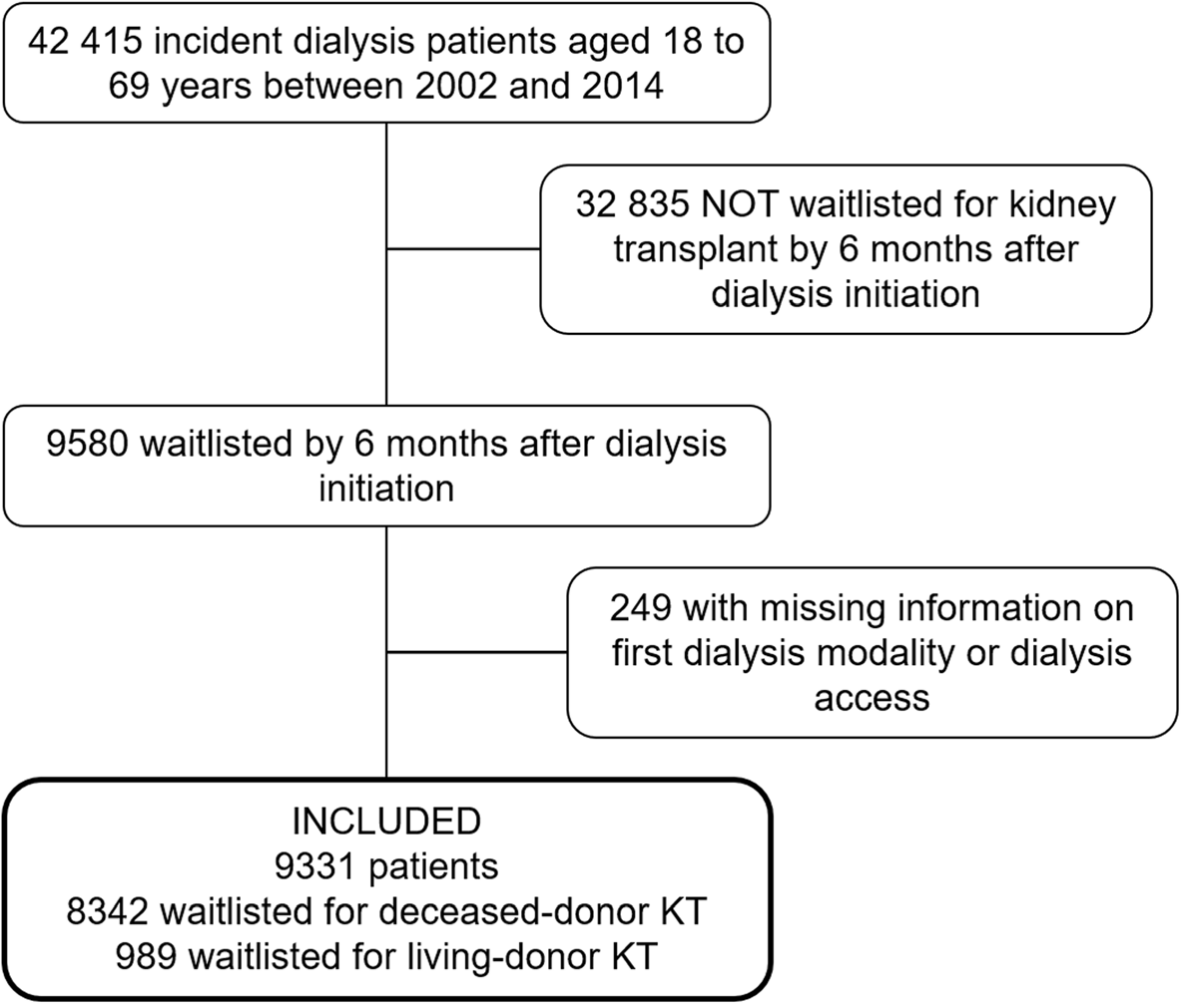
Patient selection. Abbreviations: KT, kidney transplantation

The Renal Epidemiology and Information Network (REIN) registry was set up in 2002 and includes all patients with end-stage kidney disease who start kidney replacement therapy in France [20]. The 9580 patients in this study were aged 18 to 69 years, had started hemo- or peritoneal dialysis between January 2002 and December 2014, and were placed on the national KT waiting list before or within 6 months after starting dialysis (Fig. 1). We excluded 249 patients with information missing about either first dialysis modality or first dialysis access. Patients were then classified according to whether they were wait-listed for DDKT only (*n* = 8342) or for LDKT (*n* = 989, including 490 patients simultaneously awaiting deceased donation). In France, registration on the national waiting list is mandatory for all KT candidates, for either living or deceased donation, and information about the intended donor type is routinely recorded.

The REIN registry and its utilization for research purposes have been approved by the relevant French ethics committees, specifically, the *Comité consultatif sur le traitement de l’information en matière de recherche* (CCTIRS) and the *Commission nationale de l’informatique et des libertés* (CNIL N° 903,188). French regulations do not require participants’ written or verbal informed consent for their inclusion in population-based registries requiring exhaustiveness. Patients are informed about the registration of all individuals with treated end-stage kidney disease in the REIN registry by the nephrology clinic as well as about their right to not participate (opt out).

### Information

The dialysis access strategy, defined by the combination of dialysis modality and vascular access type at hemodialysis initiation (CVC or AV access), was our main exposure variable, in three classes: hemodialysis with AV access or with CVC, and peritoneal dialysis. AV fistulae and AV grafts could not be distinguished at hemodialysis initiation, but AV graft use has been shown to be marginal in prevalent hemodialysis patients in France (about 3%) [21].

Our analyses also considered three categories of baseline variables. The first category included demographic data: age, gender, region of residence, and work status (in the labor market or not). The second category covered clinical data: primary kidney disease categorized in six groups (glomerulonephritis, diabetic nephropathy, vascular nephropathy, polycystic kidney disease, others, and unknown primary kidney disease), cardiovascular comorbidities assessed as a four-level qualitative variable (0, 1, 2, or ≥ 3 cardiovascular comorbidities among arrhythmia, coronary artery disease, congestive heart failure, and cerebrovascular disease), peripheral vascular disease, diabetes, history of previous transplantation (kidney graft excluded), and physical disabilities (autonomous versus partially or totally dependent). Nutritional status was also assessed through body mass index (categorized as < 18.5 kg/m^2^, 18.5–24.9, 25–29.9 and ≥ 30) and hypoalbuminemia (defined as serum albumin < 30 g/dl). The third category included data related to the ownership of the nephrology facility (public university centers, public non-university centers, private not-for-profit and private-for-profit centers), and whether dialysis start was planned or not. Unplanned dialysis start is defined in the registry as any first dialysis session initiated in life-threatening circumstances requiring dialysis within 24 h. Blood group (A, B, AB, and O) and panel reactive antibody level were also collected. If panel reactive antibody level was ≥85%, the patient was considered highly sensitized.

In France, any wait-listed patient who is temporary unavailable or unsuitable for KT is assigned a *temporary inactive status*. This status is also applied to patients wait-listed before a complete final check-up. These periods of inactive status were also recorded.

### Outcomes

The outcome of interest was KT by 31 December 2014, with death on the waiting list as a competing risk. Time to event was calculated from listing date or dialysis initiation, depending on whether the patient was preemptively wait-listed or not, to avoid the immortal time bias. Non-transplanted living patients were censored at the end of follow-up.

### Statistical methods

Patient characteristics at baseline were described for the two groups, distinguished by whether patients were wait-listed for deceased-only or living- (with or without deceased-) donor KT. We also described trends in the distribution of dialysis access categories between 2006 and 2014 in 11 French regions with full information over the period. Missing data (Table S1) were imputed with the fully conditional specification method [22]. In all, 20 imputed data sets were modeled separately and then combined to take the uncertainty related to the missing values into account. The imputation model included all of the variables listed in Table 1, as well as the geographic region, year of first treatment for end-stage kidney disease, and vital status at the end of follow-up. We then estimated adjusted multinomial odds ratios (ORs) for the three dialysis access strategy classes defined above and stratified by donor type, considering hemodialysis with AV access as the reference category. Models were adjusted for gender, age, region, year of dialysis initiation, occupational activity, primary kidney disease, history of diabetes, peripheral arterial disease, previous transplantation (kidney excluded), number of cardiovascular comorbidities, mobility status, body mass index, serum albumin, panel reactive antibody level, blood group, unplanned dialysis start, preemptive placement on waiting list, temporary inactive status, and ownership of the nephrology facility.

**Table 1:** Baseline characteristics of patients according to donor type at wait-listing for kidney transplantation

Characteristics	Donor type		***P***-value
	Deceased (*n* = 8342, 89%)	Living (*n* = 989, 11%)	
**Men, %**	62	64	0.211
**Age (years), median (IQR)**	51 (41–59)	45 (33–55)	< 0.001
**Primary kidney disease, %**			< 0.001
Glomerulonephritis	24	32	
Diabetic nephropathy	12	7	
Hypertensive or vascular nephropathy	11	8	
Polycystic kidney disease	21	17	
Others	22	24	
Unknown	10	12	
**Diabetes, %**	18	13	< 0.001
**Number of cardiovascular comorbidities, %**			0.003
0	83	88	
1	13	10	
2	3	2	
≥ 3	0.5	0.4	
**Peripheral arterial disease, %**	5	4	0.208
**Mobility status (partially or totally dependent), %**	2	1	0.159
**Serum albumin (g/dL), mean (SD)**	36.0 (6.4)	35.5 (6.5)	0.094
**Body mass index (kg/m), %**			0.126
< 18.5	7	8	
[18.5,25]	47	50	
[25, 30]	30	28	
≥ 30	16	14	
**Professionally active, %**	51	65	< 0.001
**Unplanned dialysis start, %**	14	17	0.005
**History of previous transplantation (kidney excluded), %**	3	2	0.620
**Preemptive placement on waiting list, %**	47	44	0.055
**Temporary inactive status, %**	49	54	0.003
**Blood group, %**			0.091
O	46	49	
A	38	34	
B	12	13	
AB	4	3	
**PRA level ≥ 85%, %**	7	7	0.661
**Ownership of nephrology facility, %**			0.661
Public non-university centre	21	20	
Public university centre (performing KT)	28	29	
Private for-profit centre	24	25	
Private not-for-profit centre	27	26	

*Abbreviations*: *PRA* panel reactive antibody, *KT* kidney transplantation

We used the cumulative incident function, with Gray’s test, to estimate rates of KT and death according to the dialysis access strategy, stratified by intended donor type. The association between dialysis access strategy and outcomes within each donor group was assessed with a multivariate Fine and Gray analysis. In the presence of competing risks, the Fine and Gray approach is the most appropriate model for prognostic research, to estimate the probabilities of an event, given patient characteristics [23]. We estimated crude and adjusted SHRs and 95% confidence intervals (95% CI). Besides age, gender, diabetes, and region (as strata), which were systematically included in adjusted models, other covariates were selected according to the Bayesian (Schwarz) Information Criterion. The proportional hazard assumption was assessed by plotting scaled Schoenfeld residuals versus rank time, by stratification, or by inclusion of a time × covariate interaction when appropriate. Because the number of deaths before KT was very low in the group awaiting LDKT, adjusted analysis of mortality was not performed for this group. *P*-values < 0.05 were considered statistically significant. Data were analyzed with SAS 9.4 (SAS Institute, Cary, NC).

## Results

### Patient’s characteristics

Patients wait-listed for LDKT were younger and more likely to be working and to have been registered with a temporary inactive status than those wait-listed for DDKT (Table 1). DDKT patients, on the other hand, had a higher prevalence of comorbidities.

### Trends in dialysis access strategy and association with donor type

**Fig. 2:**
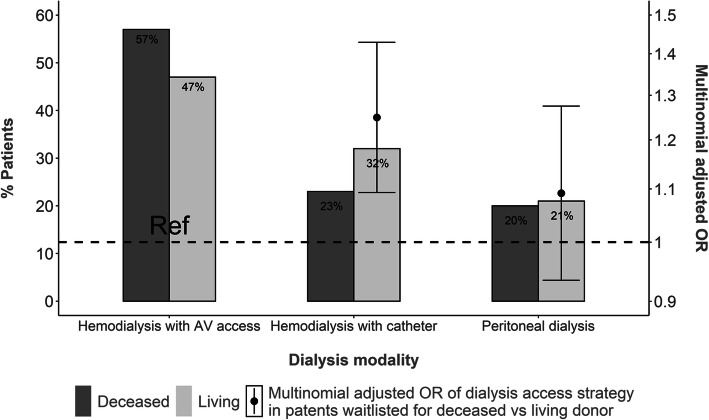
Prevalence and multinomial adjusted^#^ odds ratio of dialysis access strategy associated with donor type at placement on the kidney transplant waiting list. Abbreviations: AV, arteriovenous; OR, odds ratio. ^#^Adjusted for gender, age, region, year of dialysis initiation, occupational activity, primary renal disease, history of diabetes, peripheral arterial disease, previous transplantation (kidney excluded), number of cardiovascular comorbidities, mobility status, body mass index, serum albumin, panel reactive antibody level, blood group, unplanned dialysis start, preemptive placement on waiting list, temporary inactive status, and the ownership of the nephrology facility

**Fig. 3:**
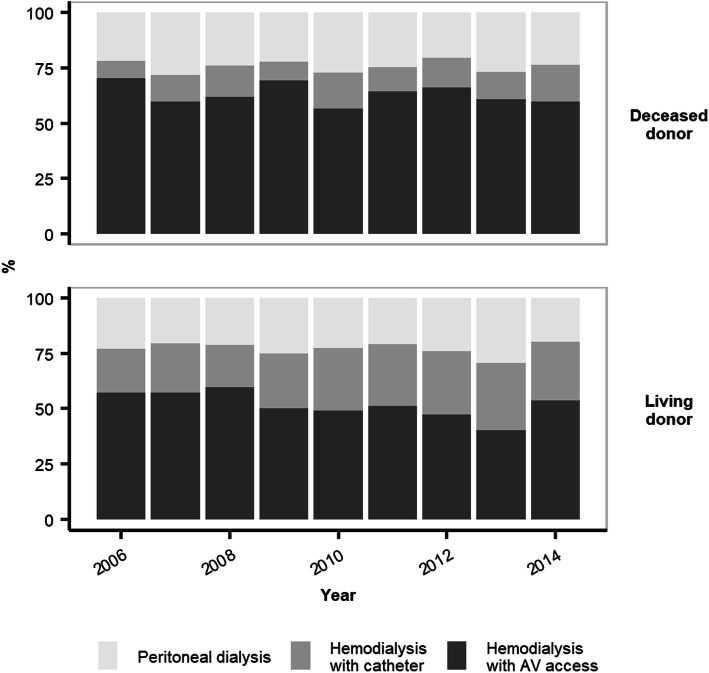
Trends in dialysis access strategy at dialysis initiation according to donor type at placement on the kidney transplantation waiting list. Abbreviation: AV, arteriovenous

Hemodialysis with AV access was the most common strategy in the study population, with a frequency higher among DDKT than LDKT patients — 57% versus 47%, respectively (Fig. 2). From 2006 through 2014, hemodialysis with CVC steadily increased in both donor groups, apparently mainly at the expense of hemodialysis with AV access (Fig. 3). In LDKT patients, hemodialysis begun with CVC regularly exceeded initial peritoneal dialysis from 2010 onward.

After adjustment for potential confounders, LDKT patients were significantly more likely than DDKT patients to start hemodialysis with a CVC than with AV access (OR 1.25, 95% CI 1.09–1.43), whereas peritoneal dialysis use did not differ between the two donor groups (OR 1.09, 95%CI 0.93–1.28). Supplementary Table S2 reports the odds ratios for each dialysis access strategy associated with other patient characteristics.

### Relation between dialysis access strategy and outcomes, by donor type

Median (interquartile range) follow-up was 43 (23–67) months. By the end of the study period, 6063 (65%) patients had undergone KT and 305 (3%) had died before KT. The number of events, the median duration of pretransplant dialysis, the cumulative incidence, and the adjusted SHRs for each outcome studied, by donor and by dialysis access strategy, are reported in Table 2. The median (interquartile range) duration of pretransplant dialysis was 15 (7–27) months for patients awaiting DDKT and 9 (5–15) months for those awaiting LDKT; it was significantly related to dialysis access strategy in both DDKT (*p*-value = 0.001) and LDKT patients (*p* = 0.003). Of note, 94% of DDKT and 87% of LDKT recipients received a graft from the expected type of donor. LDKT patients had a higher cumulative incidence of KT than DDKT patients, but this did not vary by dialysis access strategy. In DDKT patients, however, hemodialysis CVC use, compared with AV access, was associated with both a lower probability of KT and a higher probability of death before KT. No difference was observed in the cumulative incidence of either KT or mortality risk in patients with AV access and peritoneal dialysis.

**Table 2:** Number of events, time on dialysis^a^, 1- and 2-year cumulative incidence, and adjusted subdistribution hazard ratios of kidney transplantation and death before KT, by donor group

Outcome	Awaiting deceased-donor KT			Awaiting living-donor KT		
	Hemodialysis with AV access (*n* = 4752)	Hemodialysis with catheter (*n* = 1956)	Peritoneal dialysis (*n* = 1634)	Hemodialysis with AV access (*n* = 467)	Hemodialysis with catheter (*n* = 316)	Peritoneal dialysis (*n* = 206)
**Kidney transplantation**						
Number of events	3172	1157	1148	262	205	119
Median time on dialysis^a^ (IQR), months	14.8 (7.4–26.5)	15.9 (8.3–27.6)	13.6 (7.0–25.1)	8.9 (5.6–17.3)	7.5 (4.3–12.0)	9.7 (5.1–16.4)
1-year cumulative incidence (IQR), %	10 (9–10)	4 (3–4)	14 (13–16)	26 (23–30)	27 (24–30)	30 (25–36)
Adjusted^b^ SHR (95% CI)	Reference	0.88 (0.82–0.94)	1.01 (0.94–1.09)	Reference	1.18 (0.95–1.45)	0.93 (0.72–1.19)
**Death before KT**						
Number of events	149	107	44	0	3	2
Median time on dialysis^a^ (IQR), months	26.9 (11.1–41.7)	19.2 (7.6–36.5)	22.2 (15.8–37.8)	–	20.2 (16.5–26.1)	7.1 (7.1–13.4)
1-year cumulative incidence (IQR), %	5 (5–5)	14 (13–14)	6 (5–7)	–	0	0.4 (0.04–1.9)
Adjusted^c^ SHR (95% CI)	Reference	1.53 (1.14–2.04)^d^	1.12 (0.80–1.56)^d^	–	–	–

^a^In patients who had the event

^b^Subdistribution hazard ratios of kidney transplantation were adjusted for gender, age, region (as strata), year of dialysis initiation, history of diabetes, peripheral arterial disease, serum albumin, panel reactive antibody level, blood group, temporary inactive status, and ownership of the nephrology facility

^c^Subdistribution hazard ratios of death before kidney transplantation included a time interaction term for dialysis modality and were adjusted for gender, age, region (as strata), history of diabetes, peripheral arterial disease, previous transplantation (kidney excluded), number of cardiovascular comorbidities (as strata), mobility status, and serum albumin. These were not estimated for the group awaiting living-donor kidney transplant because of the small number of events

^d^Hazard ratio at 12 months of follow-up

## Discussion

This national registry-based study shows that in patients wait-listed early for KT, hemodialysis with AV access was the most common strategy, but highlights a trend towards greater use of CVC at dialysis start. LDKT were more likely to start hemodialysis with a CVC than their counterparts wait-listed for DDKT; the use of peritoneal dialysis did not differ between these groups. As expected, LDKT patients had a shorter median time on dialysis and a higher cumulative incidence of KT than DDKT patients, but this difference was not related to dialysis access strategy in the LDKT group. Inversely, in the DDKT group, CVC use was associated with a lower incidence of transplantation and a higher mortality risk than AV access.

Our findings suggest that the type of kidney donor envisioned influences vascular access strategy in patients requiring hemodialysis. While AV fistula is the most used hemodialysis access in both donor groups, some nephrologists seem to favor CVC over AV access in patients awaiting LDKT, who may undergo only short-term hemodialysis. This strategy was not, however, without risk, since patients with CVC in the DDKT group had higher mortality. We cannot draw this conclusion for the LDKT group, because of the low number of events. Our results highlight current practices in a patient situation that may represent a “gray zone”, with very little evidence about the benefit/risk ratio of different types of vascular access.

Several recent studies have challenged the Fistula First paradigm in hemodialysis patients in terms of survival [24–26], primary patency [27, 28], and cost-effectiveness [29, 30]. Findings from these studies provide further evidence in favor of a more patient-centered approach in vascular access choice, one that takes into account the likelihood of AV fistula maturation, the use of previous vascular accesses, the patient’s life expectancy, and their quality of life [31–34]. Nevertheless, attention has focused mainly on the choice between AV fistula and AV graft in the elderly, for whom catheter dependency is often an adverse outcome. Preference for an AV fistula has never or rarely ever been called into question in young adult patients with low comorbidity profiles, who might have high probability of AV fistula maturation, but also of transplantation. Woo & Lok [35] discussed the importance of an end-stage kidney disease life plan in choosing vascular access and suggested that peritoneal dialysis might be the best option for patients anticipating an LDKT. Nevertheless, our study shows that LDKT patients were not more likely than those awaiting DDKT to choose peritoneal dialysis. Hemodialysis remains the main dialysis modality in this population, and their optimal vascular access strategy has not been investigated so far.

Our study of wait-listed adults indicates that the hemodialysis duration envisioned may influence vascular access strategy. When no potential living donor is available, hemodialysis duration is expected to be longer even in patients wait-listed early for KT as in this study. Using donor type as a surrogate, we observed that patients awaiting LDKT — and thus expecting a relatively short duration of hemodialysis — were more likely to receive CVC than DDKT patients were, although the rate of peritoneal dialysis did not differ between the two donor types. These results may suggest a potential hemodialysis access strategy that allows more liberal use of CVC in patients with anticipated LDKT to preserve their vascular capital in case of kidney transplant failure. Yet, AV access was the most frequent type of vascular access in both groups, used in 47% of those wait-listed for LDKT and 57% for DDKT.

In a single-center 14-year retrospective cohort of children and adolescents, Merouani et al. [36] observed a substantial decrease in AV fistula use from 76% before the introduction of pediatric prioritization in graft allocation in Canada to 41% afterwards. Reasons for this shift from AV fistula to CVC in children, besides the high rates of KT and the switch to peritoneal dialysis, may include the difficulty in obtaining a patent fistula and the avoidance of access-related steal syndrome and congestive heart failure [36, 37]. Most of these aspects may also be relevant for adults wait-listed for LDKT. Moreover, patient reluctance about or refusal of an AV fistula may account for a significant portion of CVC use [38].

Nonetheless, although time on hemodialysis awaiting KT was, as hypothesized, shorter among LDKT than DDKT patients, these durations of pretransplant hemodialysis remained substantial. In our study, a median dialysis duration of 8 to 10 months was seen in LDKT candidates, and often more than 14 months in DDKT candidates. Hemodialysis duration may not be as short as initially expected, and prolonged CVC use may place patients at increased risk of death as they await KT. In our study, the SHR of death associated with CVC in the DDKT group was 1.53 (95%CI 1.14–2.04).

In a recent cohort study, Kim et al. [39] hypothesized that a short dialysis period before LDKT would yield results similar to those of preemptive KT. Their purpose was also to validate a cutoff value for duration of pretransplant dialysis to differentiate clinical outcomes. After evaluating the outcomes of LDKT according to this duration in both unmatched and propensity-score-matched cohorts, they found no difference in either mortality or death-censored graft survival between recipients of preemptive and non-preemptive KT who had pretransplant dialysis durations < 19 months. Patient survival was worse, however, when pretransplant dialysis duration was 19 months or longer in a propensity-score-matched LDKT cohort. On the other hand, Goto et al. [40] demonstrated that preemptive KT was associated with a reduced rate of clinical events including mortality, graft failure and post-transplant cardiovascular disease (3.3%), while this event rate was significantly higher among those with a pretransplant dialysis duration < 1 year (10.8%). Similarly, Prezelin-Reydit et al. [41] recently showed that preemptive KT was strongly associated with a lower hazard of graft failure than pretransplant dialysis (HR 0.57 95%CI 0.51–0.63), regardless of its duration — even only 6 months. Their study did not consider the type of vascular access.

Our findings are consistent with the extensive literature on vascular access outcomes, which points toward an association between CVC use and poor outcomes. CVC has been associated with higher risk of mortality [26, 42] and of infectious and non-infectious complications [26, 43] than either AV fistula or graft. It has also been shown to be the least cost-effective vascular access for hemodialysis when either the burden of hospitalization [29] or quality of life [30] is considered. These results from observational studies, taken together, have formed the basis of current vascular access guidelines and the Fistula First, Catheter Last Initiative. Although the impact of residual confounding on assessment of vascular access outcomes is a subject of growing concern [24, 26, 30], the vascular access strategy in our population of KT candidates is unlikely to be driven by short life expectancy. Nevertheless, the relative survival benefit associated with AV fistula in our study should be seen in the perspective of the very low mortality rates of patients wait-listed for KT. Other outcomes related to vascular access strategy, such as access function, morbidity, quality of life, and cost-effectiveness, should be evaluated in this population [44, 45].

Major strengths of this study include the size and unselected nature of our registry-based population. We adjusted our analysis for several potential confounders that might affect either vascular access choice or access to KT, such as unplanned dialysis start, panel reactive antibody level, and temporary inactive status. Some of these factors were also independent predictors of mortality. We also applied an alternative method that takes into account the competing risk of death before transplantation. Conventional methods such as the Kaplan-Meier method and standard Cox proportional hazards regression may be inappropriate in the presence of competing risks, which may hinder the observation of the event of interest. In this case, an adjusted subdistribution hazards approach has been shown to be most appropriate for prognostic research [23].

Our study also has limitations. First, due to the observational nature of the study, the results can only describe associations; causality cannot be inferred. Second, LDKT accounts for highly variable portions of KT among countries, and national allocation policies may have a substantial impact on waiting-list time. Our results may thus be specific to certain contexts. Third, the small number of adverse events in the LDKT group highlights the need for dedicated cohort studies focused on dialysis access for these patients.

## Conclusions

Our study shows that vascular access strategy differs according to whether hemodialysis patients were wait-listed for living- or deceased-donor KT. Although AV fistula was the most frequently used dialysis access in the study population, some nephrologists tended to favor CVC over AV fistula to start hemodialysis in patients awaiting living donation, likely because they expect the duration of dialysis to be short. Although this strategy may protect these patients from AV fistula creation and its potential drawbacks, known complications of CVC (such as infections and thrombosis) we indicate the need for caution. Given the difficulty of predicting pretransplant hemodialysis duration, the risk of complications from long-term CVC use must be carefully evaluated. More studies are required to clarify the benefit/risk ratio of dialysis access strategy for non-fatal outcomes among KT candidates.

## Supplementary information

**Additional file 1: Table S1.** Percentage of missing data among study covariates. **Table S2.** Factors associated with the choice of hemodialysis with catheter or peritoneal dialysis rather than hemodialysis with arteriovenous access in patients wait-listed early for kidney transplantation - Multinomial logistic regression.

## Data Availability

The restrictions due to French Personal data protection regulation (CNIL) prohibit the authors from making the minimal data set publicly available. The access to the data of the REIN registry is governed by a charter. It implies the approval by the REIN scientific board which analyses each request. Information about the data of the REIN registry can be requested by mail to Dr. Christian Jacquelinet who manages the REIN registry at the French Biomedicine Agency (christian.jacquelinet@biomedecine.fr).

## Abbreviations

AV: Arteriovenous
CI: Confidence intervals
CVC: Central venous catheter
DDKT: Deceased-donor kidney transplantation
ESKD: End-stage kidney disease
KT: Kidney transplantation
LDKT: Living-donor kidney transplantation
OR: Odds ratio
SHR: Subdistribution hazard ratio
