# Supplementary material for: Practice patterns of dialysis access and outcomes in patients wait-listed early for kidney transplantation
Source: BMC Nephrol. 2020 Oct 2;21:422. doi: 10.1186/s12882-020-02080-5 (PMC7532567; doi:10.1186/s12882-020-02080-5)
Supplement: Supplementary file 1 — Additional file 1: Table S1. Percentage of missing data among study covariates. Table S2. Factors associated with the choice of hemodialysis with catheter or peritoneal dialysis rather than hemodialysis with arteriovenous access in patients wait-listed early for kidney transplantation - Multinomial logistic regression. [file 12882_2020_2080_MOESM1_ESM.docx]

**Practice patterns of dialysis access and outcomes in patients wait-listed early for kidney transplantation**

**Supporting information**

**Table S1**. Percentage of missing data among study covariates.

**Table S2.** Factors associated with the choice of hemodialysis with catheter or peritoneal dialysis rather than hemodialysis with arteriovenous access in patients wait-listed early for kidney transplantation - Multinomial logistic regression

**Table S1**. Percentage of missing data among study covariates.

| **Characteristics** | **Missing (%)** |
| --- | --- |
| Gender | 0 |
| Age | 0 |
| Primary kidney disease | 0 |
| Diabetes | 1.7 |
| Number of cardiovascular comorbidities | 2.5 |
| Peripheral arterial disease | 2.8 |
| Mobility status (partially or totally dependent) | 13.1 |
| Serum albumin ≥ 30 g/dL | 40.7 |
| Body mass index (kg/m) | 24.1 |
| Professional status | 22.8 |
| Unplanned dialysis start | 4.8 |
| History of previous transplantation (kidney excluded) | 9.2 |
| Preemptive placement on waiting-list | 0 |
| Temporary inactive status | 0 |
| Blood group | 0 |
| Panel reactive antibody level ≥ 85% | 0 |
| Ownership of nephrology facility | 0.4 |

**Table S2**: Factors associated with the choice of hemodialysis with catheter or peritoneal dialysis rather than hemodialysis with arteriovenous access in patients wait-listed early for kidney transplantation - Multinomial logistic regression.

| **Characteristics** | **Odds ratios (95% confidence intervals)*** | | **p** |
| --- | --- | --- | --- |
|  | **Hemodialysis**  **with catheter** | **Peritoneal**  **dialysis** |  |
| **Women** | 1.07 (0.98- 1.16) | 1.36 (1.24- 1.50) | <0.001 |
| **Age (years)** | 0.99 (0.98- 0.99) | 0.99 (0.99- 1.00) | <0.001 |
| **Primary kidney disease, %** |  |  | <0.001 |
| Glomerulonephritis | ref | ref |  |
| Diabetic nephropathy | 1.07 (0.90- 1.27) | 1.04 (0.83- 1.31) |  |
| Hypertensive or vascular nephropathy | 1.54 (1.35- 1.75) | 0.85 (0.72- 0.99) |  |
| Polycystic kidney disease | 0.62 (0.53- 0.71) | 0.49 (0.42- 0.56) |  |
| Others | 1.25 (1.11- 1.40) | 0.89 (0.78- 1.01) |  |
| Unknown | 1.53 (1.33- 1.76) | 1.03 (0.87- 1.21) |  |
| **Diabetes, %** | 1.01 (0.88- 1.16) | 0.77 (0.64- 0.92) | 0.01 |
| **Number of cardiovascular comorbidities, %** |  |  | 0.2 |
| 0 | ref | ref |  |
| 1 | 1.09 (0.98- 1.22) | 1.01 (0.88- 1.15) |  |
| 2 | 1.18 (0.97- 1.43) | 1.08 (0.83- 1.40) |  |
| ≥3 | 1.47 (0.97- 2.25) | 0.82 (0.42- 1.62) |  |
| **Peripheral arterial disease, %** | 0.79 (0.67- 0.93) | 0.90 (0.73- 1.11) | 0.02 |
| **Mobility status (partially or totally dependent), %** | 1.47 (1.16- 1.85) | 0.98 (0.67- 1.44) | 0.003 |
| **Serum albumin ≥ 30 g/dL, %** | 0.93 (0.93- 0.94) | 1.02 (1.01- 1.03) | <0.001 |
| **Body mass index (kg/m), %** |  |  | <0.001 |
| <18.5 | 1.05 (0.89- 1.26) | 0.95 (0.77- 1.18) |  |
| [18.5,25[ | ref | ref |  |
| [25,30[ | 0.98 (0.88- 1.08) | 0.93 (0.84- 1.04) |  |
| ≥30 | 0.90 (0.80- 1.02) | 0.70 (0.61- 0.80) |  |
| **Professional occupation, %** | 1.01 (0.91- 1.11) | 1.61 (1.44- 1.79) | <0.001 |
| **Unplanned dialysis start, %** | 15.0 (13.6- 16.6) | 0.92 (0.75- 1.13) | <0.001 |
| **History of previous transplantation (kidney excluded), %** | 1.21 (0.93- 1.57) | 0.67 (0.45- 0.98) | 0.02 |
| **Temporary inactive status, %** | 1.06 (0.97- 1.15) | 0.77 (0.70- 0.85) | <0.001 |
| **Blood group, %** |  |  | 0.004 |
| O | ref | ref |  |
| A | 1.08 (1.00- 1.18) | 0.97 (0.89- 1.07) |  |
| B | 1.26 (1.12- 1.41) | 0.98 (0.85- 1.13) |  |
| AB | 1.14 (0.94- 1.38) | 0.87 (0.69- 1.11) |  |
| **PRA level ≥ 85%, %** | 1.17 (1.01- 1.35) | 0.88 (0.73- 1.06) | 0.02 |
|  |  |  |  |
| **Ownership of nephrology facility, %** |  |  | <0.001 |
| Public non-university centre | 0.71 (0.64- 0.79) | 1.16 (1.02- 1.32) |  |
| Public university centre (performing KT) | ref | ref |  |
| Private for-profit centre | 0.51 (0.46- 0.56) | 0.24 (0.20- 0.29) |  |
| Private not-for-profit centre | 0.33 (0.29- 0.38) | 2.89 (2.54- 3.29) |  |

Abbreviations: PRA, panel reactive antibody; KT, kidney transplantation.

*Odds ratios of either hemodialysis with catheter or peritoneal dialysis, compared with hemodialysis with arteriovenous access
